# Aberrant dynamic properties of whole‐brain functional connectivity in acute mild traumatic brain injury revealed by hidden Markov models

**DOI:** 10.1111/cns.14660

**Published:** 2024-03-05

**Authors:** Liyan Lu, Fengfang Li, Hui Li, Leilei Zhou, Xinying Wu, Fang Yuan

**Affiliations:** ^1^ Department of Radiology, Nanjing First Hospital Nanjing Medical University Nanjing Jiangsu China; ^2^ Department of Radiology, Nanjing Drum Tower Hospital The Affiliated Hospital of Nanjing University Medical School Nanjing China; ^3^ Department of Neurosurgery, Shanghai Jiao Tong University Affiliated Sixth Peoples' Hospital, School of Medicine Shanghai Jiao Tong University Shanghai China

**Keywords:** acute mild traumatic brain injury, cognitive impairment, dynamic functional connectivity, hidden Markov model, resting‐state functional magnetic resonance imaging, transition probability

## Abstract

**Objectives:**

This study aimed to investigate the temporal dynamics of brain activity and characterize the spatiotemporal specificity of transitions and large‐scale networks on short timescales in acute mild traumatic brain injury (mTBI) patients and those with cognitive impairment in detail.

**Methods:**

Resting‐state functional magnetic resonance imaging (rs‐fMRI) was acquired for 71 acute mTBI patients and 57 age‐, sex‐, and education‐matched healthy controls (HCs). A hidden Markov model (HMM) analysis of rs‐fMRI data was conducted to identify brain states that recurred over time and to assess the dynamic patterns of activation states that characterized acute mTBI patients and those with cognitive impairment. The dynamic parameters (fractional occupancy, lifetime, interval time, switching rate, and probability) between groups and their correlation with cognitive performance were analyzed.

**Results:**

Twelve HMM states were identified in this study. Compared with HCs, acute mTBI patients and those with cognitive impairment exhibited distinct changes in dynamics, including fractional occupancy, lifetime, and interval time. Furthermore, the switching rate and probability across HMM states were significantly different between acute mTBI patients and patients with cognitive impairment (all *p* < 0.05). The temporal reconfiguration of states in acute mTBI patients and those with cognitive impairment was associated with several brain networks (including the high‐order cognition network [DMN], subcortical network [SUB], and sensory and motor network [SMN]).

**Conclusions:**

Hidden Markov models provide additional information on the dynamic activity of brain networks in patients with acute mTBI and those with cognitive impairment. Our results suggest that brain network dynamics determined by the HMM could reinforce the understanding of the neuropathological mechanisms of acute mTBI patients and those with cognitive impairment.

## Graphical abstract

Alteration in switching frequency and transition probability between acute mTBI and HC. Alteration in switching frequency and transition probability between mTBI patients with and without cognitive impairment.

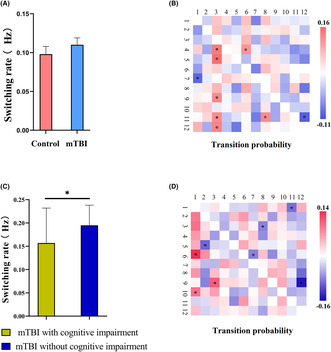

## 1 INTRODUCTION

Traumatic brain injury (TBI) is a major cause of morbidity and mortality and a substantial burden on public health services worldwide.^1^, ^2^ Mild TBI (mTBI) accounts for 58%–88% of all TBIs, according to epidemiologic studies.^3^ The diagnostic criteria for mTBI include no or short‐lived loss of consciousness (LOC) (less than 30 min), no posttraumatic amnesia (PTA) or PTA lasting less than 24 h, and a Glasgow Coma Scale (GCS) score of 13–15 upon presentation to the emergency department.^4^ Clinically, approximately 25% of mTBI patients suffer from persistent neurocognitive sequelae and symptoms while most individuals with mTBIs recover quickly and spontaneously.^5^ However, mTBI as defined above is not associated with demonstrable abnormalities on conventional computed tomography (CT) or magnetic resonance imaging (MRI), which makes it difficult to characterize neurophysiology for patient identification and the development of individualized therapy.^5^ Consequently, novel objective techniques revealing the underlying neuropathological mechanisms in acute mTBI patients and those with cognitive impairment are urgently needed.

In recent decades, resting‐state functional magnetic resonance imaging (rs‐fMRI) has been widely utilized to study abnormal brain activity in individuals with brain disorders, including mTBI.^6^, ^7^, ^8^, ^9^ Numerous rs‐fMRI studies have identified disrupted functional connectivity (FC) in patients with mTBI and cognitive impairment.^7^, ^10^, ^11^, ^12^ However, the study of FC relies on the assumption that resting‐state FC is “stationary” over the course of the scan.^13^ However, this assumption, which ignores a large degree of variability in FC during the rs‐fMRI, may be outdated. Increasing evidence suggests that the human brain system is complexly dynamic and that FC fluctuates over time during scans.^13^, ^14^, ^15^, ^16^ Several studies have reported aberrant dynamical characterizations, such as the dynamics of FC in patients with mTBI or cognitive impairment.^17^, ^18^, ^19^, ^20^, ^21^ Therefore, studying the dynamics of brain connectivity as a new research direction in the field of neuroimaging may be crucial for revealing the pathophysiological mechanisms underlying acute mTBI and cognitive impairment.

The sliding window approach has been most commonly utilized to study alterations in brain dynamics.^22^ Utilizing the sliding window approach, abnormalities in the default mode network (DMN), somatomotor network (SMN), and salience network (SN) have been frequently identified in our previous rs‐fMRI studies and other studies involving mTBI patients.^17^, ^19^, ^21^ Moreover, we previously found that abnormal brain activity in the SN and ventral attention network (vAN) was correlated with Montreal Cognitive Assessment (MoCA) scores and could be used as a clinical diagnostic biomarker and a marker of disease severity.^10^ However, the sliding window approach has several limitations.^23^, ^24^, ^25^ The time scale of the window size, including the width of the window and the length of the step, is predefined.^26^ The selection of the optimal window size is crucial because a window that is too long will limit the visualization of fast dynamics, whereas a window that is too short will miss sufficient data for reliable network estimation.^27^ A hidden Markov model (HMM) could overcome the main limitation of the selection of the optimal window size.^26^, ^28^ This model has been used to describe brain activity as a dynamic sequence of discrete brain states directly assessed from resting data.^28^ Previous studies have shown that the HMM is capable of capturing the dynamics of brain activity on minimal time scales.^28^ In addition, previous studies have confirmed that quick changes in brain activity are far from random; therefore, an HMM could help to provide a richer description of the dynamic nature of brain activity in central nervous system disease on short timescales.^26^, ^28^

In this study, we present an HMM analysis of rs‐fMRI data acquired from participants with acute mTBI and healthy controls (HCs). We aimed to explore the complicated temporal dynamics of brain activity and to characterize specific patterns of transitions and brain networks across states in both acute mTBI patients and patients with cognitive impairment.

## 2 MATERIALS AND METHODS

### 2.1 Participants

Of the 221 patients who visited the emergency department between August 2019 and October 2021, 80 patients with mTBI were willing to participate in this study. In addition, 65 HCs were enrolled from the local community. The inclusion criteria for the mTBI patients were as follows^29^: (1) a GCS score ranging from 13 to 15 at presentation to the emergency department; and (2) a closed head injury within 14 days with either an LOC shorter than 30 min or a PTA shorter than 24 h. The exclusion criteria for all participants were as follows: (1) a history of brain injury; (2) a history of neurological disease or long‐standing psychiatric conditions; (3) MRI contraindications; and (4) coexisting alcohol and drug abuse. This prospective study was approved by the institutional review board of Nanjing Medical Hospital. All participants provided written informed consent before undergoing an MRI.

### 2.2 Cognitive assessment

All participants underwent neuropsychological assessment via the Beijing version of the MoCA, a tool suggested for the assessment of cognitive impairment following mTBI. Although this screening tool is simple, changes in the MoCA score can be not only statistically significant but also clinically relevant. The MoCA is sufficiently stable and sensitive to detect the early presence of cognitive impairment after mTBI and to assess changes over time.^30^ This test takes approximately 10 min to administer and has a maximum score of 30 points. A score greater than 26 was considered to indicate normal cognition, while a score less than 26 was considered to indicate cognitive impairment. All participants completed the MoCA test within 12 h of the MRI.

### 2.3 Magnetic resonance imaging acquisition

A 3.0T MRI scanner (Ingenia, Philips Medical Systems, Netherlands) equipped with a 12‐channel head coil was used. Parallel imaging was employed. For each participant, rs‐fMRI data and high‐resolution T1‐weighted anatomic imaging data were acquired. rs‐fMRI data were obtained using a gradient echo‐planar imaging sequence (axial: repetition time [TR] = 2000 ms; echo time [TE] = 30 ms; slices = 36; thickness = 4 mm; gap = 0 mm; field of view [FOV] = 240 mm × 240 mm; acquisition matrix = 64 × 64; flip angle [FA] = 90°; and total volume = 230). The fMRI sequence took 8 min and 8 s. High‐resolution T1‐weighted anatomic imaging data were acquired with a three‐dimensional turbo fast echo (3D‐TFE) T1‐weighted imaging sequence (T1WI) (sagittal: TR = 8.1 ms; TE = 3.7 ms; FA = 8°; FOV = 256 mm × 256 mm; acquisition matrix = 256 × 256; thickness = 1 mm; gap = 0 mm; and total slices = 172). Fluid attenuated inversion recovery (FLAIR) was performed with the following parameters: TR = 7000 ms; TE = 120 ms; slices = 18; thickness = 6 mm; gap = 1.3 mm; FA = 110°; and voxel size = 0.65 mm × 0.95 mm × 6 mm. Susceptibility weighted imaging (SWI) was performed with a 3D gradient echo (GRE) sequence with the following parameters: TR = 22 ms; TE = 34 ms; FA = 20°; acquisition matrix = 276 × 319; thickness = 1 mm; and FOV = 220 mm × 220 mm. All participants were instructed to stay awake, keep their heads still, and keep their eyes closed. Foam padding was used to reduce head motion. No participants reported falling asleep during the scan when asked immediately after the scan.

### 2.4 Magnetic resonance imaging data preprocessing

The rs‐fMRI data were preprocessed using Graph Theoretical Network Analysis (GRETNA) software (http//www.nitrc.org/projects/gretna) implemented in MATLAB (version R2013b). Standard preprocessing methods were used.^31^ First, the first 10 volumes were discarded due to the instability of the initial magnetic field. Second, slice timing was performed for the remaining functional images, and head motion was corrected. No group differences were observed with respect to head motion (*p* > 0.05). Then, functional images were spatially coregistered with their high‐resolution T1‐weighted anatomic images with a voxel size of 3 mm × 3 mm × 3 mm^3^. Next, rs‐fMRI time series were bandpass filtered (0.01–0.08 Hz) to improve the signal‐to‐noise ratio. Afterward, the normalized data were smoothed with a 3‐mm full width at half maximum (FWHM) Gaussian kernel. Finally, to remove spurious sources of variance, the Friston 24 parameter was obtained via head motion correction, and signals from cerebrospinal fluid, white matter, and the whole brain were regressed.

### 2.5 Hidden Markov model

**FIGURE 1:**
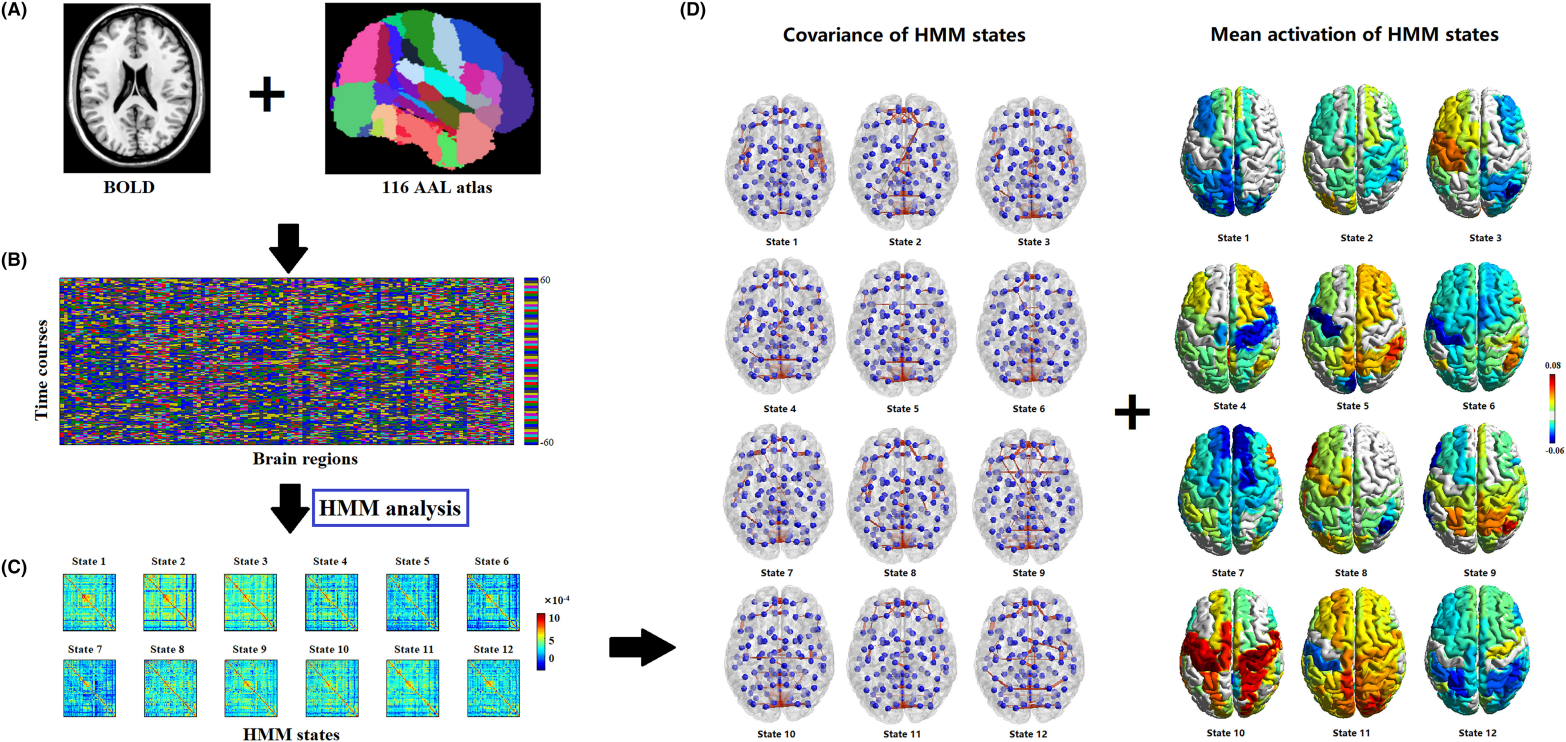
Schematic workflow with a hidden Markov model (HMM). (A) The whole‐brain regions were parcellated into 116 regions of interest (ROIs) based on the Automated Anatomical Labeling (AAL) atlas, and the time courses were extracted by averaging the BOLD signals across voxels within each ROI for each participant. (B) Data on the time course of 116 brain regions × 220 time points were acquired for each subject. (C) The HMM analysis was run on the time courses, and an HMM with 12 states was obtained. (D) Each HMM state was characterized as a multivariate Gaussian distribution including a covariance matrix and a mean activity.

The HMM, a Markov process involving unobtained (hidden or latent) states, was applied as described in Wang et al.^27^ First, based on the Automated Anatomical Labeling (AAL) atlas, whole‐brain regions were parcellated into 116 regions of interest (ROIs) for each participant (Figure 1A). Second, time course data for 116 regions × 220 time points were acquired for each subject (Figure 1B). Third, the number of HMM states was evaluated by summary statistics, including the minimum free energy and medial fractional occupancy across the HMM states; ultimately, 12 states were identified, which was consistent with the findings of one previous study^27^ (Figure 1C). Each state was modeled as a multivariate normal distribution, which included first‐order (mean activity) and second‐order statistics (covariance matrix) statistics (Figure 1D). The HMM analysis identified periods of quasi‐stationary activity, where the 116 ROI time courses could be described by specific configurations of mean activity and FC.

### 2.6 Analysis of the dynamics and transitions of hidden Markov model states

According to the inferred HMM states, a range of properties that could effectively reflect the within‐subject temporal dynamics were estimated.^27^, ^32^ These properties were as follows: (1) Fractional occupancy of HMM states, computed as the temporal proportions of active HMM state; (2) lifetime (s), computed as the amount of time spent in a specific state; (3) interval time (s), computed as the amount of time between consecutive visits to a state; (4) switching rate (Hz), indicating the switching frequency for each HMM state; and (5) transition probability matrix, the core metric of the HMM, representing the probability of transition between all pairs of HMM states.

### 2.7 Statistical analysis

The SPSS 23.0 software package (SPSS, Inc., Chicago, IL, USA) was used in this study. Continuous data are shown as the mean ± standard deviation (SD), whereas categorical variables are presented as counts. The Shapiro‐Wilk (SW) test was used to assess the data distribution. Independent sample *t* tests were used for continuous variables, and chi‐square tests or Fisher's exact tests were used for categorical variables. The fractional occupancy, lifetime, interval time, and switching rate of HMM states were tested using two‐tailed two‐sample *t* tests between acute mTBI patients and HCs and between acute mTBI patients with cognitive impairment and those without cognitive impairment. A false discovery rate (FDR) correction was applied for multiple comparisons. A threshold of *p* < 0.05 was considered to indicate statistical significance.

The transition probabilities of HMM states between acute mTBI patients and HCs and between acute mTBI patients with cognitive impairment and those without cognitive impairment were investigated utilizing nonparametric permutation testing.^27^ A total of 5000 permutations were performed across patients with mTBI and HCs to effectively generate a null distribution of differences in the global dynamics of each state between groups. The *p* values were subsequently computed.

Spearman's correlation analysis was performed to analyze the associations between the differences in fractional occupancy, lifetime, and interval time of HMM states and MoCA scores in acute mTBI patients with cognitive impairment. *p* < 0.05 was set as the threshold and was corrected for age, sex, and education level.

## 3 RESULTS

### 3.1 Demographic and clinical data

**FIGURE 2:**
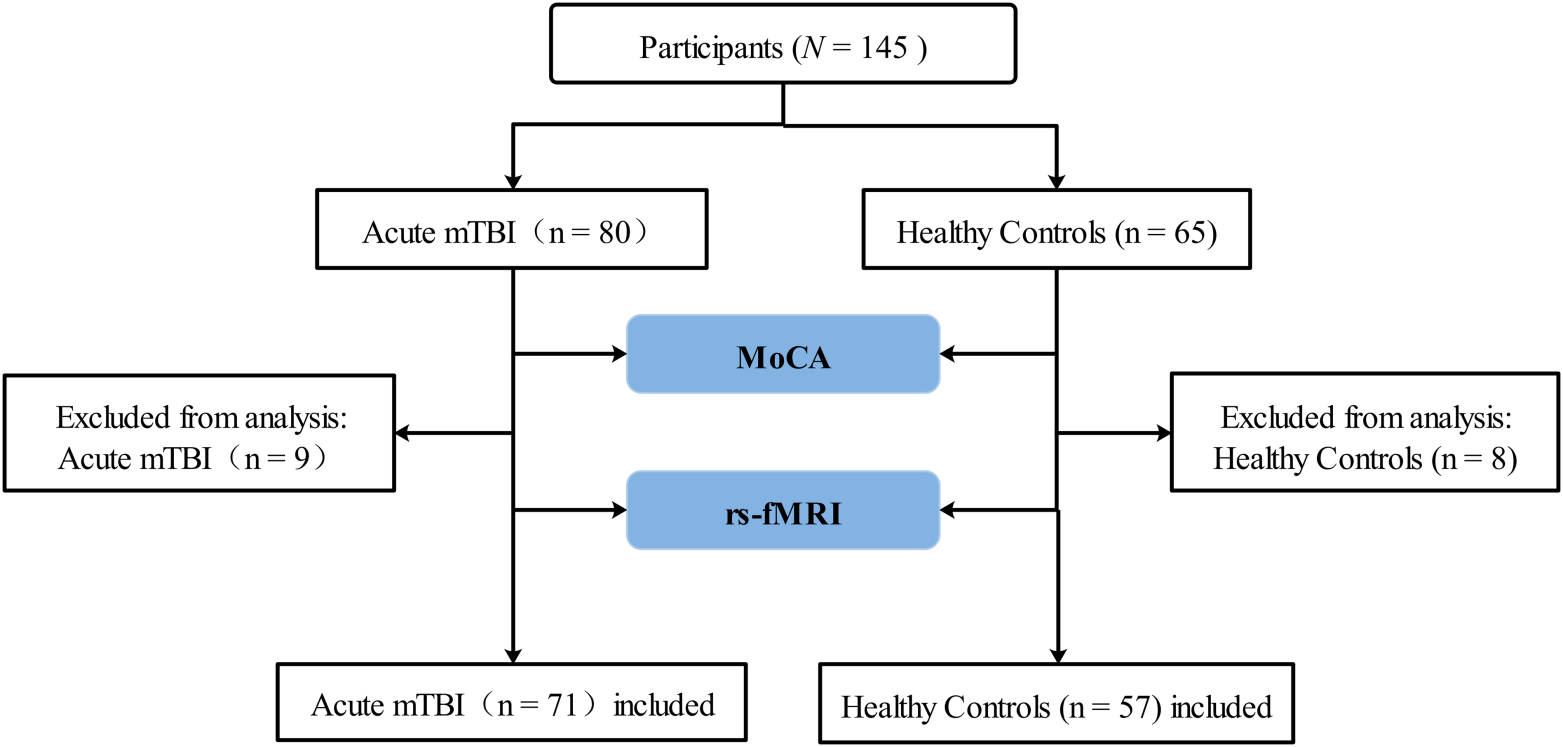
Flow chart of participant enrollment.

Among the 80 acute mTBI patients, nine patients were excluded because of preexisting neurological or psychiatric disease (*n* = 1), previous head injury (*n* = 3), image distortion (*n* = 2), or head motion (*n* = 3). Among the 65 HCs, 8 patients were excluded because of preexisting neurological or psychiatric disease (*n* = 1), previous head injury (*n* = 4), dental appliance (*n* = 1), or head motion (*n* = 2) (Figure 2). Thus, data from 71 acute mTBI patients and 57 HCs were ultimately analyzed in this study. As shown in Table 1, at the emergency department, all patients with acute mTBI had an initial GCS of 15. There were no significant differences in terms of age (*p* = 0.125), sex (*p* = 0.101), or education (*p* = 0.128) between the acute mTBI group and the HC group. MR images were obtained at an average of 4.3 days (range, 0–11 days) after head injury in 71 patients. MoCA scores, which represent cognitive performance, were also recorded and evaluated (Table 1). There was a significant difference in MoCA scores between acute mTBI patients and HCs (*p* < 0.001). Moreover, there were significant differences in age (*p* = 0.011), education (*p* < 0.001), and MoCA scores (*p* < 0.001) between the acute mTBI patients with cognitive impairment and those without cognitive impairment but no significant difference in sex (*p* = 0.901) (Table 2). FLAIR and SWI showed no visible traumatic lesions. During the study period, no acute mTBI patients took medications because of mild symptoms.

**TABLE 1:** Summary of demographic characteristics between acute mTBI patients and healthy controls.

Characteristics	mTBI (*n* = 71)	HC (*n* = 57)	*p* value
Age (years)	43.54 ± 10.43	46.16 ± 8.79	0.125
Sex (male/female)	32/39	34/23	0.101
Education (years)	11.46 ± 3.95	12.39 ± 2.85	0.128
GCS score	15	–	
Time since injury (days)	4.39 ± 2.40	–	
MoCA	24.54 ± 2.83	27.37 ± 2.17	<0.001*

*Note*: The data are presented as means ± standard deviations.

Abbreviations: GCS, Glasgow Coma Scale; MoCA, Montreal Cognitive Assessment; mTBI, mild traumatic brain injury.

* *p* < 0.05.

**TABLE 2:** Summary of demographic characteristics between acute mTBI patients with and without cognitive impairment.

Characteristics	Cognitive impairment (*n* = 41)	Without cognitive impairment (*n* = 30)	*p* value
Age (years)	46.20 ± 9.96	39.90 ± 10.10	0.011*
Sex (male/female)	17/24	12/18	0.901
Education (years)	9.95 ± 4.07	13.53 ± 2.68	<0.001*
GCS Score	15	15	1.000
Time since injury (days)	4.45 ± 2.13	4.32 ± 2.51	0.872
MoCA	22.68 ± 2.22	27.07 ± 1.05	<0.001*

*Note*: The data are presented as means ± standard deviations.

Abbreviations: GCS, Glasgow Coma Scale; MoCA, Montreal Cognitive Assessment; mTBI, mild traumatic brain injury.

* *p* < 0.05.

### 3.2 Aberrant dynamics for each hidden Markov model state in acute mild traumatic brain injury patients and correlation analysis

**FIGURE 3:**
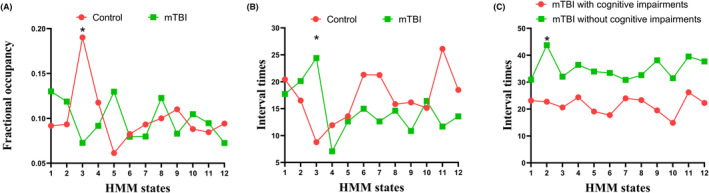
(A) Significant differences in fractional occupancies in each state between patients with acute mTBI and HCs. (B) The significant difference in the interval times of each state between acute mTBI patients and HCs. Red represents HCs, and green represents mTBI patients. (C) There was a significant difference in the interval times of each state between acute mTBI patients with cognitive impairment and those without cognitive impairment. Red represents acute mTBI patients with cognitive impairment, and green represents acute mTBI patients without cognitive impairment. All temporal properties were evaluated using a two‐tailed, two‐sample *t* test. *Significant group differences (*p* < 0.05).

**FIGURE 4:**
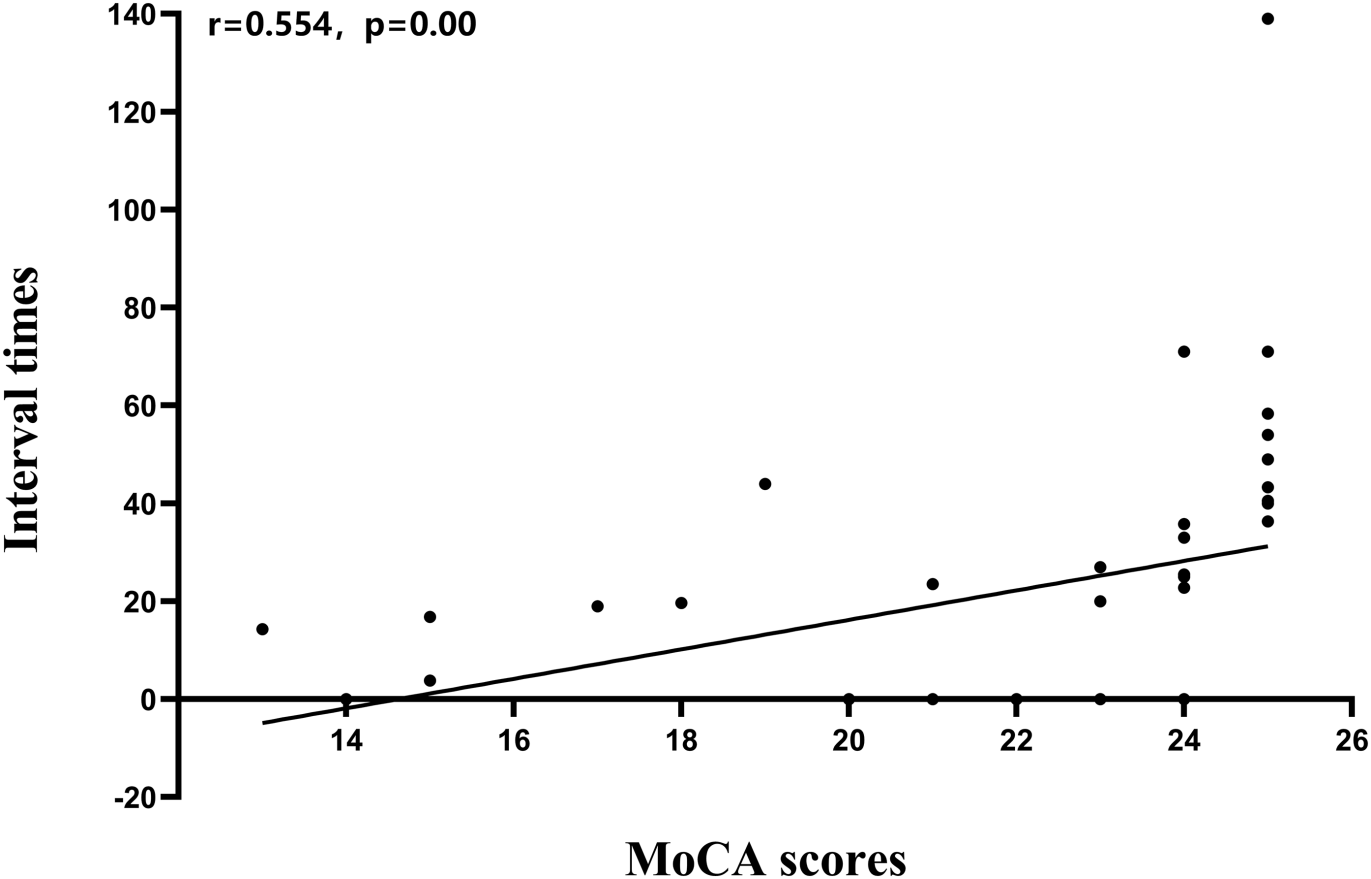
Statistically significant correlations between the interval times of state 2 and the MoCA score in acute mTBI patients with cognitive impairment. A Spearman correlation analysis was performed. *p* < 0.05 was considered to indicate statistical significance. The interval time of HMM state 2 was significantly positively correlated with the MoCA score (*r* = 0.554, *p* = 0.00) in acute mTBI patients with cognitive impairment.

In addition to the data distribution, the time courses of the visits to each of the brain states were also estimated with the HMM inference. In the HMM, fractional occupancies were used to explore the temporal characteristics of acute mTBI. Compared with that of HCs, the fractional occupancy of HMM state 3 in acute mTBI patients was significantly lower (*p* = 0.001) (Figure 3A). The fractional occupancies of the remaining HMM states displayed no significant differences between acute mTBI patients and HCs (Figure 3A). The interval time of HMM state 3 was significantly greater for participants with acute mTBI (*p* = 0.005) (Figure 3B), and the interval time of HMM state 2 was significantly lower for acute mTBI patients with cognitive impairment (*p* = 0.005) (Figure 3C). However, the lifetimes of all the HMM states between acute mTBI patients and controls and the fractional occupancies and lifetimes of all the HMM states between acute mTBI patients with and without cognitive impairment were not significantly different (all *p* > 0.05). Our results revealed unique patterns of temporal reorganization of brain microstates in patients with acute mTBI and cognitive impairment over a short time scale. Moreover, for acute mTBI patients with cognitive impairment, the interval time of HMM state 2 was significantly positively correlated with the MoCA score (*r* = 0.554, *p* = 0.00) (Figure 4).

### 3.3 Aberrant transition patterns between hidden Markov model states

**FIGURE 5:**
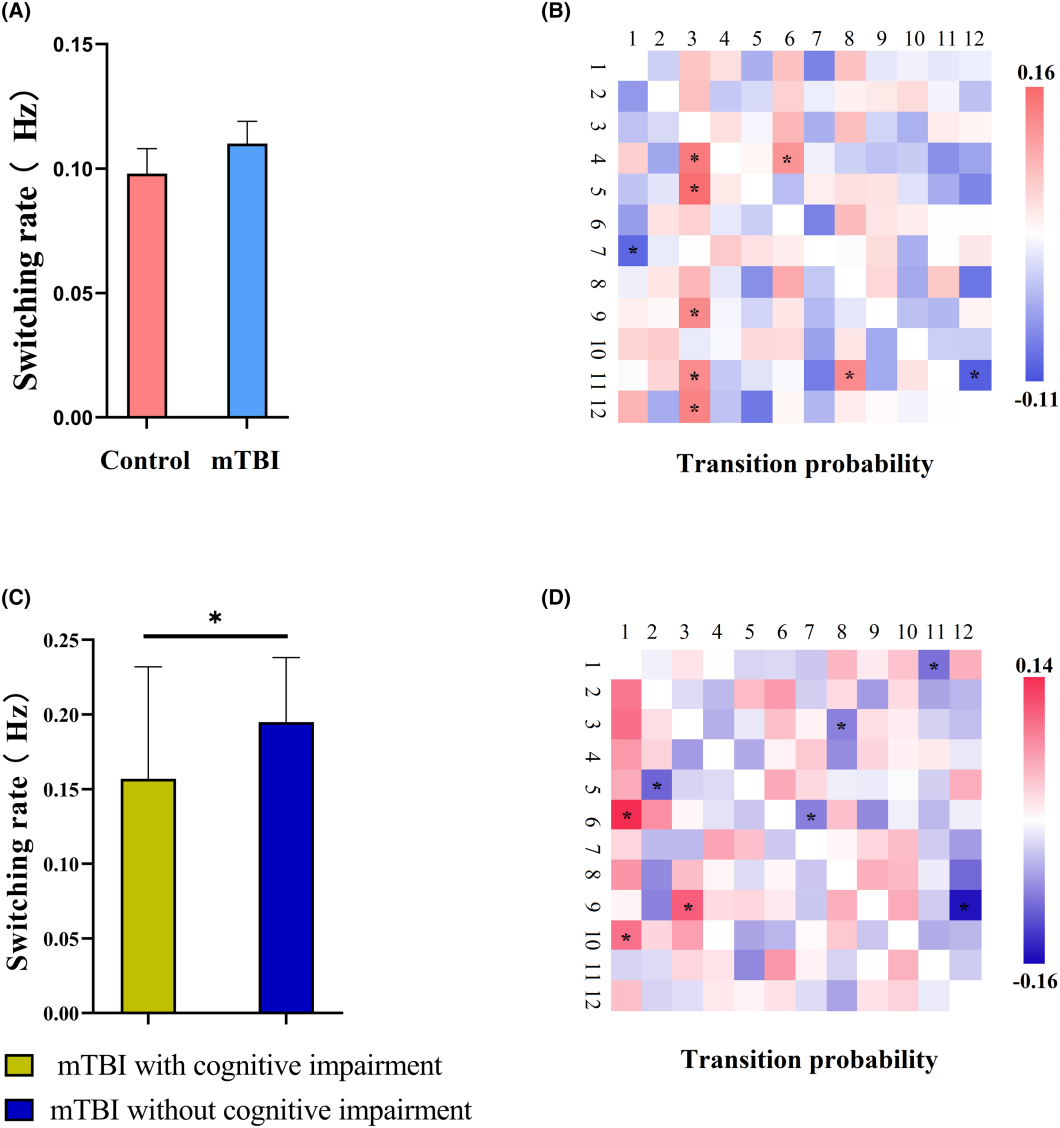
(A) Alteration in switching frequency between acute mTBI patients and HCs. (B) The significant alteration in transition probability between acute mTBI patients and HCs. Red represents a significant increase in acute mTBI patients compared with HCs, and blue represents a significant decrease in acute mTBI compared with HCs. (C) Alteration in switching frequency between mTBI patients with and without cognitive impairment. (D) The significant alteration in transition probability between acute mTBI patients with cognitive impairment and those without cognitive impairment. Red represents a significant increase in acute mTBI patients with cognitive impairment compared with acute mTBI patients without impairment, and blue represents a significant decrease in acute mTBI patients with cognitive impairment compared with acute mTBI patients without cognitive impairment. Significant group differences were evaluated using a permutation test with 5000 permutations. *p < 0.05.

The switching rate was not significantly different between acute mTBI patients and HCs (*p* = 0.379) (Figure 5A), suggesting that acute mTBI patients and HCs had similar stable network dynamic patterns during the entire scan process. Furthermore, we compared the transition probabilities of HMM states between acute mTBI patients and HCs using permutation analysis (5000 permutations) in order to investigate the different transition probabilities of the HMM states. Significant group differences in switching probability between HMM states are shown in Figure 5B. Compared to those of HCs, the switching probabilities from HMM states 4, 5, 9, 11, and 12 to state 3, from state 7 to state 1, from state 11 to state 8, and from state 4 to state 6 increased significantly for acute mTBI (state 4 to 3: *p* = 0.0296; state 5 to 3: *p* = 0.0264; state 9 to 3: *p* = 0.0376; state 11 to 3: *p* = 0.0406; state 12 to 3: *p* = 0.0332; state 7 to 1: *p* = 0.0456; state 11 to 8: *p* = 0.0354; and state 4 to 6: *p* = 0.0496). The switching probability from HMM state 11 to state 12 significantly decreased in patients with acute mTBI (*p* = 0.0360). These results suggest significant aberrant transition patterns between HMM states in acute mTBI patients.

The switching rate significantly differed between acute mTBI patients with cognitive impairment and without cognitive impairment (0.16 ± 0.07 vs. 0.19 ± 0.04, *p* = 0.009) (Figure 5C), suggesting that acute mTBI patients with and without cognitive impairment had different network dynamic patterns during the entire scan. Furthermore, we compared the transition probability of HMM states between acute mTBI patients with and without cognitive impairment. Significant group differences in switching probability between HMM states are shown in Figure 5D. Compared to those of acute mTBI patients without cognitive impairment, the switching probabilities from HMM states 6 and 10 to state 1, and from state 9 to state 3 increased significantly for acute mTBI patients with cognitive impairment (state 6 to 1: *p* = 0.00; state 10 to 1: *p* = 0.02; and state 9 to 3: *p* = 0.01). The switching probabilities from HMM state 1 to state 11, from state 3 to state 8, from state 5 to state 2, from state 6 to state 7, and from state 9 to state 12 significantly decreased for acute mTBI patients with cognitive impairment (state 1 to 11: *p* = 0.03; state 3 to 8: *p* = 0.04; state 5 to 2: *p* = 0.02; state 6 to 7: *p* = 0.04; state 9 to 12: *p* = 0.00). These results suggest significant aberrant transition patterns between HMM states in acute mTBI patients with cognitive impairment.

### 3.4 Brain activation maps of states

**FIGURE 6:**
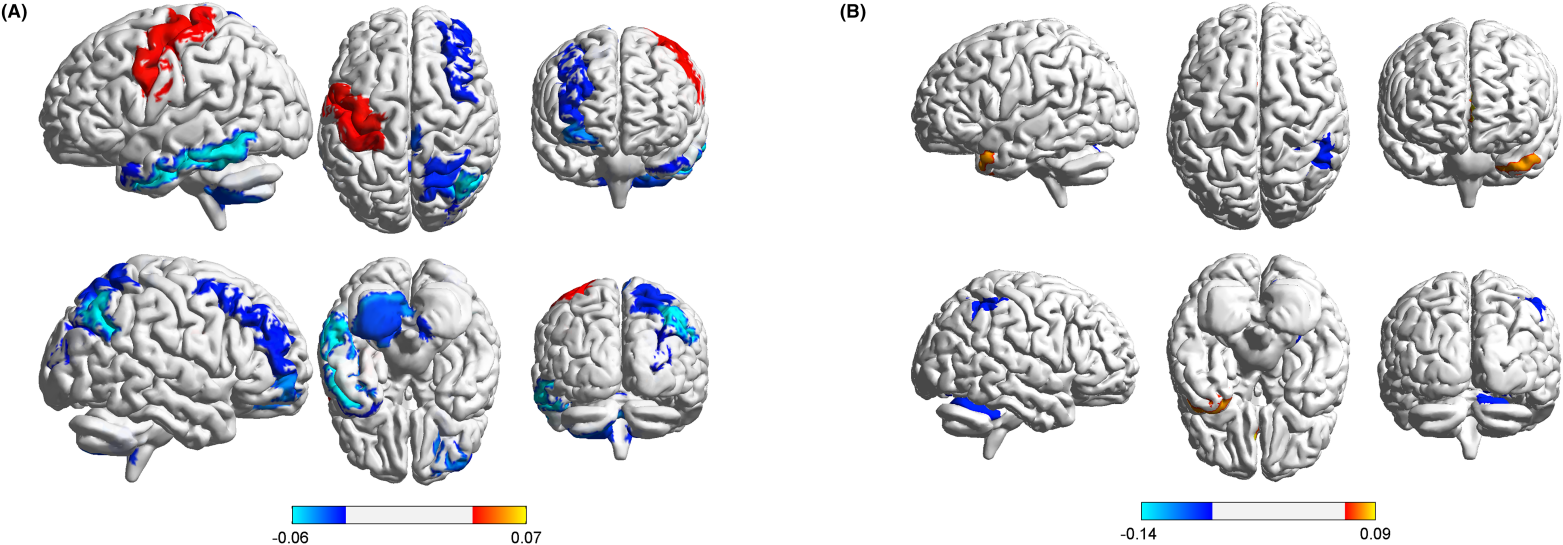
(A) Mean activation distribution of state 3 mainly induced by acute mTBI. (B) Mean activation distributions of state 2 mainly induced by acute mTBI with cognitive impairment.

The spatial activation maps of the large‐scale whole‐brain network states in acute mTBI patients were dominated by state 3 (Figure 6A). The main increases in HMM state 3 were observed in the right precentral gyrus and the decreases occurred in DMN areas (including the left superior and medial frontal gyri), left postcentral gyrus, subcortical areas (including the left bilateral angular gyrus), left superior occipital gyrus, right superior middle temporal gyrus, supplementary motor areas, and cerebellum. The spatial activation maps of the large‐scale whole‐brain network states in acute mTBI patients with cognitive impairment were dominated by state 2 (Figure 6B). HMM state 2 demonstrated the majority of increased activation in the superior middle temporal gyrus and decreased activation in the postcentral gyrus and cerebellum.

## 4 DISCUSSION

In this study, we tested the hypothesis that aberrant changes in the dynamic brain functional network occur in acute mTBI patients and in those with cognitive impairment; however, previous studies have not fully captured these changes. The results suggest that individuals with acute mTBI and those with cognitive impairment exhibit temporal brain reconfiguration, as shown through a combination of rs‐fMRI and HMM analyses. The HMM could provide a better temporal description than those of previous studies using the sliding window approach.

Resting‐state functional magnetic resonance imaging can be used to characterize dynamic connectivity in individuals with mTBI and cognitive impairment.^17^, ^18^, ^19^, ^20^, ^21^ The HMM is a useful methodology, and HMM analysis can represent brain states and transitions.^23^ Previous studies have indicated that FC in the human brain is highly dynamic.^13^, ^25^, ^32^ In this study, 12 HMM states for brain activity were identified in patients with acute mTBI and HCs.^27^ The time course of visits to each HMM state of the brain was subsequently analyzed, and temporal brain reconfiguration was found in patients with acute mTBI and those with cognitive impairment. Our results demonstrated that, compared with HCs, acute mTBI patients exhibited significant differences in overall brain states. Our previous work utilizing dynamic FC showed that acute mTBI patients exhibited increased fractional occupancy and mean lifetime for the states, which were characterized by high modularity.^33^, ^34^ The lifetime and fractional occupancy of the brain states are crucial for explaining the neurological mechanisms underlying psychiatric illnesses.^23^, ^35^, ^36^ Unfortunately, no correlations between these aberrations and neurocognitive performance were observed. The present study revealed that acute mTBI patients exhibited significantly decreased fractional occupancies and significantly increased interval times in the HMM states. Consistent with previous findings, these alterations suggested that acute mTBI patients had high modularity and strong connections between brain areas, which may be compensatory responses induced by mTBI.^33^, ^34^, ^36^ Furthermore, acute mTBI patients with cognitive impairment had significantly decreased interval times of the HMM states, and the interval time of HMM state 2 was significantly positively correlated with the MoCA score. This could be because acute mTBI patients with cognitive impairment exhibit hypoconnectivity in brain regions. This inconsistency of results may be attributed to the potential impact that different methods have on reported group differences and remains a limitation within this upcoming field. Our study provides comprehensive information about the temporal features and promising biomarkers for acute mTBI patients and those with cognitive impairment.^27^, ^36^

The human brain is a complex dynamic system. It transitions smoothly and continuously through states to directly support cognitive performance. Understanding these transitions is critical for our understanding of functional brain plasticity in patients with acute mTBI and those with cognitive impairment. The switching rate across all 12 HMM states, which was defined as the frequency of transitions between states, was analyzed. The results showed that there were no significant differences in the switching rate of brain states between acute mTBI patients and HCs. Our results suggest that acute mTBI patients and HCs exhibit similar patterns of transitions across brain connectivity. However, significant group differences in switching probability between HMM states were found, suggesting that the transition pattern between brain states was significantly altered in acute mTBI patients. The switching rate and probability were significantly different between acute mTBI patients with and without cognitive impairment, suggesting that acute mTBI patients with cognitive impairment exhibit an inflexible pattern of transitions. More specifically, HMM analysis could capture most of the information contained in the data.

Based on the spatial activation maps of the large‐scale whole‐brain network states induced by acute mTBI, our results indicate hypoactivation in the DMN, subcortical network, SMN, VN, auditory network (AUDN), and cerebellum network (CN), and hyperactivation in the SMN. Based on the spatial activation maps of the large‐scale whole‐brain network states induced by acute mTBI with cognitive impairment, our results indicate the hypoactivation in the SMN and CN, and hyperactivation in the AUDN. These findings are consistent with previous findings that patients with acute mTBI exhibit aberrant static and dynamic network interactions in the DMN, VN, SMN, and AUDN.^19^, ^37^, ^38^, ^39^ Furthermore, reduced dynamic functional network connectivity (dFNC) in patients with acute mTBI was correlated with neurocognitive performance.^19^, ^37^ The DMN, VN, SMN, and AUDN are widely studied networks in mTBI patients. These networks show an aberrant connectivity across a wide pathophysiological spectrum of acute mTBI. Notably, we observed that hypoactivation in the CN in both acute mTBI patients and those with cognitive impairment, which was consistent with the findings of one previous study. The CN, which is not often studied, was captured in this study. To date, only a few studies have demonstrated that acute mTBI patients have abnormal fractional anisotropy in the cerebellum, and altered cerebellar fractional anisotropy is associated with cognitive impairment.^37^, ^40^ Taken together, these findings show that the CN is also important in acute mTBI and may be a promising biomarker for studying acute mTBI and related cognitive impairment. Reduced FNC reflects network dysfunction, and increased FNC is attributed to a compensatory mechanism or reorganization of the network after microstructural damage to the brain.

In this study, patients were evaluated immediately after injury, but whether dynamic aberrations might be temporary disturbances remains an open question. This possibility should be addressed in future longitudinal works to determine whether dynamic aberrations are always present during semiacute or even chronic states. Although that, in conclusion, this study is the first to investigate dynamic alterations in acute mTBI patients and acute mTBI patients with cognitive impairment using HMM analysis. Compared with HCs, patients with acute mTBI had altered brain dynamics. Moreover, dynamic aberrations were observed in patients with acute mTBI with cognitive impairment. Our findings reveal reorganizations of brain states at various time scales and multiple transition patterns in patients with acute mTBI and cognitive impairment. These findings provide new insights into the pathophysiological mechanism of brain injury and cognitive impairment after acute mTBI.

## FUNDING INFORMATION

This work was supported by the National Natural Science Foundation of China (No. 82102012, No. 82102006).

## CONFLICT OF INTEREST STATEMENT

The authors declare no conflicts of interest.

## Data Availability

The data that support the findings of this study are available from the corresponding author upon reasonable request.
